# Modeling Many-Body Interactions in Water with Gaussian Process Regression

**DOI:** 10.1021/acs.jpca.4c05873

**Published:** 2024-10-11

**Authors:** Yulian T. Manchev, Paul L. A. Popelier

**Affiliations:** Department of Chemistry, The University of Manchester, Manchester M13 9PL, U.K.

## Abstract

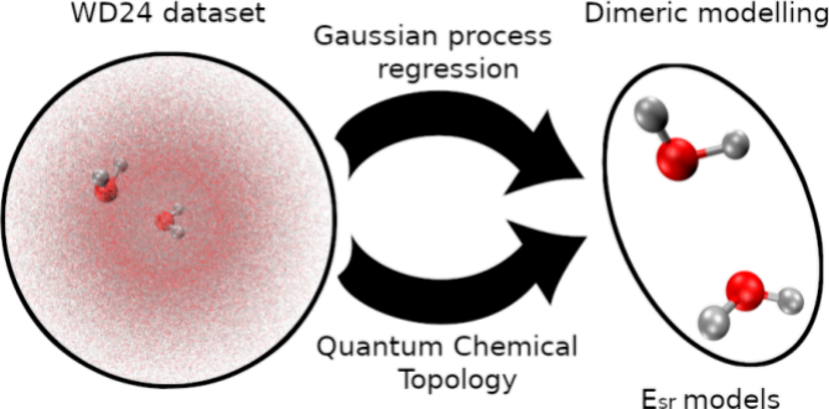

We report a first-principles water dimer potential that captures many-body interactions through Gaussian process regression (GPR). Modeling is upgraded from previous work by using a custom kernel function implemented through the KeOps library, allowing for much larger GPR models to be constructed and interfaced with the next-generation machine learning force field FFLUX. A new synthetic water dimer data set, called WD24, is used for model training. The resulting models can predict 90% of dimer geometries within chemical accuracy for a test set and in a simulation. The curvature of the potential energy surface is captured by the models, and a successful geometry optimization is completed with a total energy error of just 2.6 kJ mol^–1^, from a starting structure where water molecules are separated by nearly 4.3 Å. Dimeric modeling of a flexible, noncrystalline system with FFLUX is shown for the first time.

## Introduction

There have been tremendous efforts to accurately model the water potential energy surface (PES) by “first-principles” models. In particular, it has been shown that many-body effects play a critical role in water, and therefore they must be included to obtain accurate simulations.^1−5^ For liquid water, convergence of the many-body expansion is reached when all interactions up to and including 4-body are taken into account.^6^ There have been many proposed water potentials, which model many-body effects through the many-body expansion, with each *N*-body (where *N* is a molecule) interaction being modeled by a separate model. Popular potentials include CC-pol,^7−9^ WHBB,^10−12^ HBB2-pol,^13^ and most recently q-AQUA^14,15^ and MB-pol,^16−18^ with the latter four models being based on permutationally invariant polynomials (PIPs). The most recent models, q-AQUA and MB-pol(2023),^18^ include 4-body interactions, and both are trained on virtually the same CCSD(T) data sets. For long-range 2-body interactions, the MB-pol(2023) switches the PIPs to electrostatics and dispersion models, where the Coulomb interactions are calculated by geometry-based point charges aimed at approximating the monomer’s dipole moment. MB-pol(2023) uses a modified version of the TTM4-F^19^ empirical model. The q-AQUA potential has recently made use of the TTM3-F^20^ model for adding many-body polarization effects.^21^ MB-pol in particular has become a very popular water potential due to its high accuracy compared to other models^22^ and because it can reproduce important properties of water.^23−37^ Other water models have also been created with the Gaussian approximation potential (GAP)^38^ framework and neural networks (NNs). It has been shown that the three aforementioned machine learning (ML) methods (PIPs, GAPs, NNs) have very similar predictive performance for water clusters.^39^

## Methods

Here we briefly outline our approach for building *N*-body potentials and present a water dimer potential that does not use the many-body expansion. Instead, the PES is modeled directly by learning quantum-mechanically accurate atomic energies, as obtained through the Quantum Chemical Topology (QCT)^40,41^ and Interacting Quantum Atoms (IQA)^42^ frameworks. We then test the accuracy of the dimer potential with the next-generation ML force field FFLUX.^43^

**Figure 1:**
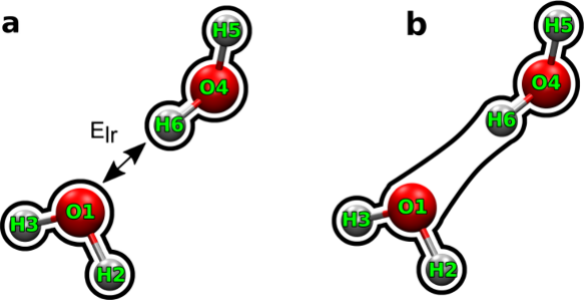
Differences between (a) monomeric versus (b) dimeric modeling within FFLUX. The black lines around the molecules show the boundary of short-range interactions. Any interactions outside of the model boundary are considered long-range, shown by the double-header arrow.

Figure 1 shows how short-range and long-range interactions change by upgrading from monomeric to dimeric modeling. Monomeric modeling refers to the case where atomic models capture interactions happening inside a single water molecule, while in dimeric modeling the atomic models also capture intermolecular interactions between two water molecules. Note that the nomenclature of short- and long-range should not be confused with that in the prevailing literature on intermolecular forces. In the latter, short-range interaction typically refers to intermolecular exchange-repulsion or dispersion (given its high inverse-power-of-a-distance behavior). Additional discussion of the interaction definitions can be found in Section 3 of the Supporting Information (SI).

Monomeric modeling has already been used successfully for condensed phase water^44^ and formamide crystal^45^ simulations with FFLUX. Hydrogen bonding in these simulations was captured by high-order multipolar electrostatics, which we showed are predicted well through GPR models.^46^ The monomeric simulations of water have shown that FFLUX can perform close to other state-of-the-art force fields, and when it does not perform well, it is due to the known limitations of monomeric modeling. The use of monomeric modeling does capture intramolecular polarization inside each water monomer but inherently lacks intermolecular polarization. Moving to dimeric modeling and beyond addresses this issue directly because then intermolecular interactions and many-body polarization effects are inherently included in the models. Dispersion effects can also be captured by the dimeric models, if the quantum mechanical calculations include them, which eliminates the need for all previous approximations of these effects, such as Lennard-Jones parameters. Therefore, there is a need to move to dimeric, and eventually *N*-meric modeling, where intermolecular interactions between *N* molecules are included in the atomic models.

**Figure 2:**
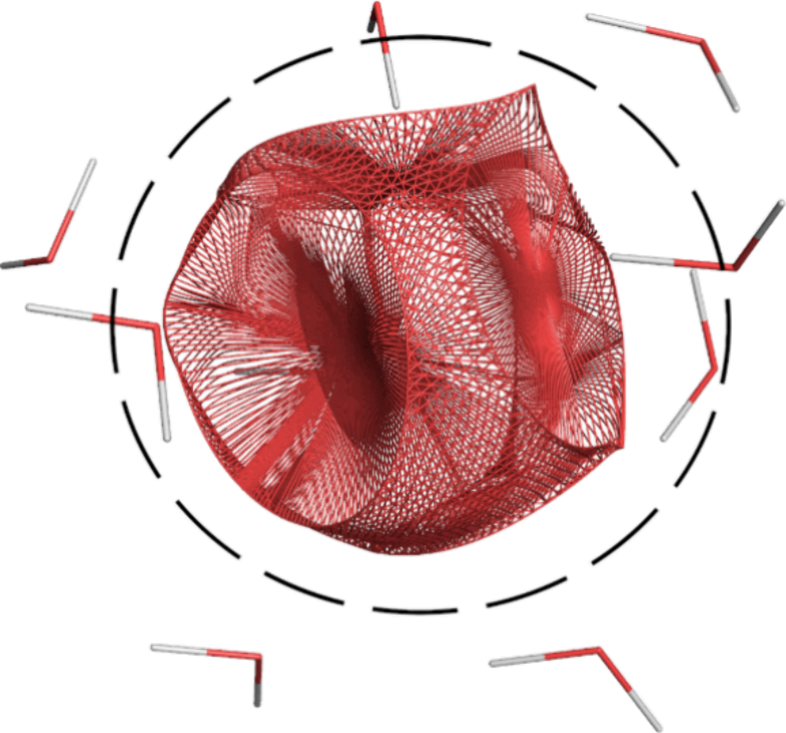
Example using *N*-meric modeling in FFLUX. The encircled region around an example water molecule (for which only the atomic volume of the oxygen atom is shown) represents the short-range interactions that will directly affect the IQA energy and multipole moments of the oxygen atom. The volume of the oxygen atom itself is distorted by the presence of nearby atoms. Interactions outside of the region are considered long-range and are calculated by multipolar electrostatics based on the atomic multipole moments. Each atom in the simulation will have its own encircled region, and the environment in this region will directly affect the atom’s IQA energy and multipole moments.

With FFLUX, any larger system can be described through individual atomic contributions, and the method is applicable to any system, not just water. Figure 2 highlights how *N*-meric modeling of many-body interactions will be captured by FFLUX simulations. The local environment of the atom, captured by the encircled region, will directly affect the atomic IQA energies and multipole moments. Short-range interactions within the encircled region are described through the IQA energy. Long-range interactions outside of the encircled region are handled through high-order multipolar electrostatics. It is also important to note that FFLUX uses flexible multipole moments, which change as the environment of an atom changes, and thus many-body polarization effects are directly expressed into the atomic multipole moments. Capturing more of the atom’s environment would directly lead to more representative atomic energies and multipole moments, and therefore more accurate simulations.

The ML method of choice in this work is exact Gaussian process regression (GPR)^47^ which is performed through the GPyTorch^48^ library, which is built on top of the popular PyTorch^49^ library. The use of the PyTorch library means that GPU-accelerated computing and automatic differentiation are natively supported during GPR model creation. The model architecture has been discussed in detail in our previous work,^50^ and the overall model architecture remains largely the same here. The notable upgrade is the use of a custom KeOps^51^ kernel function for GPR covariance matrix calculations. The KeOps library performs highly efficient on-the-fly reduction operations, such as matrix-vector multiplications, making it possible to scale exact Gaussian processes to much larger training set sizes without having to partition the covariance matrix. This allows for much larger ML models to be interfaced with FFLUX for the first time. GPR models used within FFLUX are no longer limited by the number of training points but rather by the amount of quantum mechanical data that is available. The Atomic Local Frame (ALF) descriptors^52^ (i.e., ML features) are used as model inputs. The ALF descriptors are minimal in the sense that they represent the chemical system exactly, without any redundancies, and are applicable to any chemical system.

A set of water dimer geometries that adequately sample the water dimer configuration space must be used for ML model construction. Here we create a new artificial water dimer data set consisting of 100,000 water dimer geometries with separations anywhere between 2.0 and 5.5 Å between the two water molecules. Following customary naming tradition by material abbreviation and proposal year, we refer to this data set as WD24. Additional details of the data set generation process, along with a visualization of the data set, can be found in Section 4 of the SI. The goal of the new data set is to sample the water dimer configuration space uniformly and fully. A uniform distribution of geometries is critical to ensure that each molecule in the dimer can act as both a donor and acceptor. A poorly balanced data set will alter the predictive accuracy of the GPR models. Note that the level of theory used in this proof-of-concept study was B3LYP/aug-cc-pVTZ. B3LYP calculations do show a slight systematic error of 2-body energies when compared to BSSE-corrected CCSD(T) calculations^53^ but our proposed method does not make use of the many-body expansion and we work with atomic energy partitioning instead. For the development stage of FFLUX, a lower level of theory is justified because it significantly speeds up data set generation times. While B3LYP does not include dispersion effects, it has recently been shown that these effects can be added to the atomic energies by empirical corrections.^54^

There are three reasons for constructing an artificial data set instead of obtaining dimer geometries from MD simulations, which is how geometries in ML force field data sets are typically obtained. First, the geometries obtained from simulation depend on the underlying MD method used to perform the simulation. Second, the extensive hydrogen bonding present in bulk water simulations heavily skews the possible water dimer geometries that can be acquired to ones where a hydrogen bond is present between the water molecules. Third, higher-energy water dimer geometries, which do not contain hydrogen bonds, must be included in the data set to increase the GPR model robustness.

**Figure 3:**
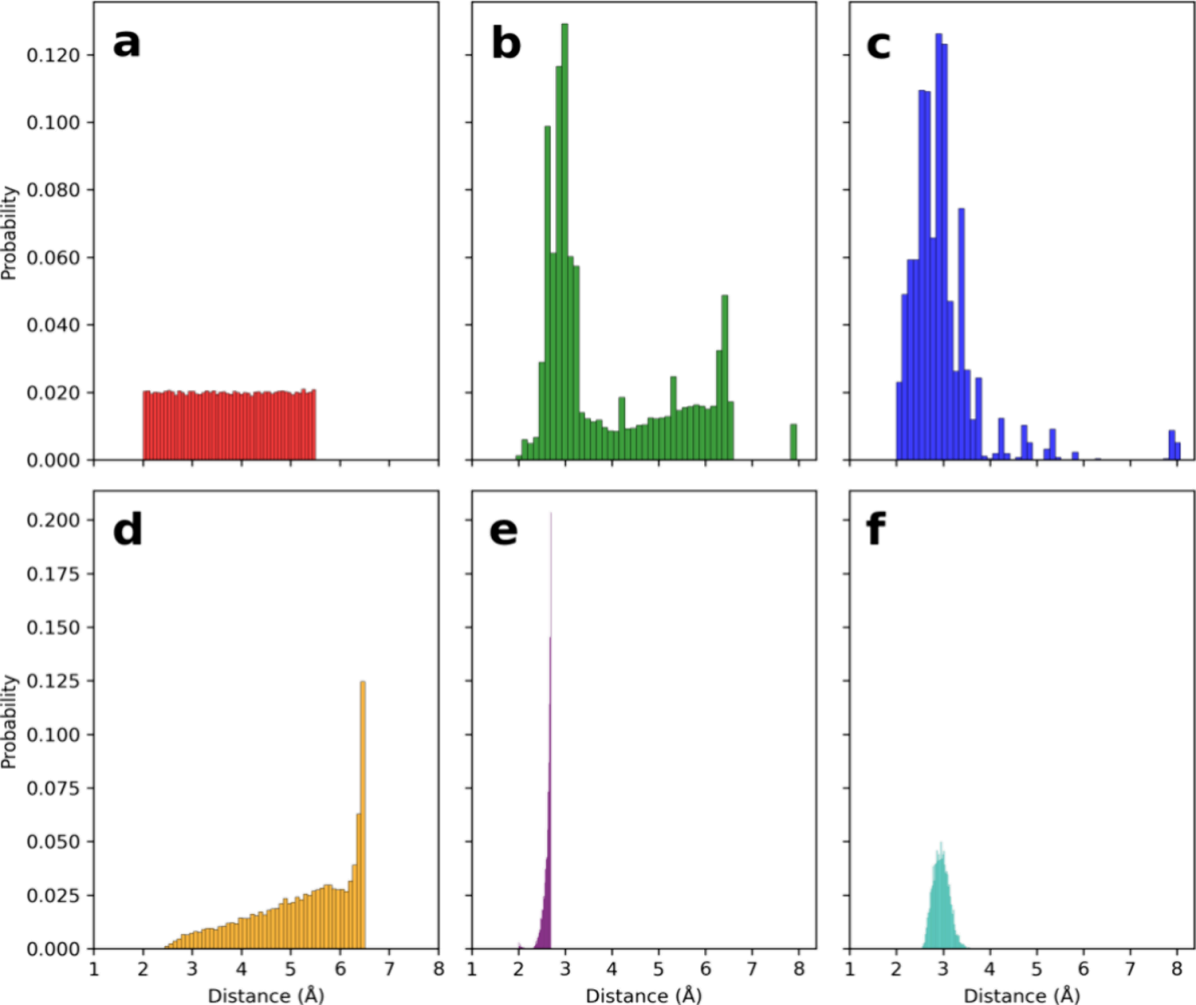
Comparison of the oxygen–oxygen distance distribution in the (a) WD24, (b) q-AQUA,^15^ and the four data sets used to fit MB-pol^16^: (c) CC-pol, (d) pimd-run105, (e) pimd-run107, and (f) stationary water dimer data sets.

Figure 3 compares the generated WD24 data set to other available water dimer data sets such as ones used to train the MB-pol and q-AQUA potentials. MB-pol uses a combination of four data sets^16^ for fitting its short-range interactions: (i) the CC-pol data set, (ii and iii) two data sets obtained from path integral molecular dynamics (PIMD) simulations using the HBB2 potential, and (iv) a data set consisting of 10 water dimer stationary points and their neighborhoods. As can be seen from the probability distributions, other data sets unevenly sample the configuration space. The WD24 data set attempts to sample all regions as uniformly as possible, such that performance of the GPR models is similar across all sampled space. Angular distributions, which show similar trends, are found in Figures S2 and S3 of the SI.

## Results and Discussion

**Figure 4:**
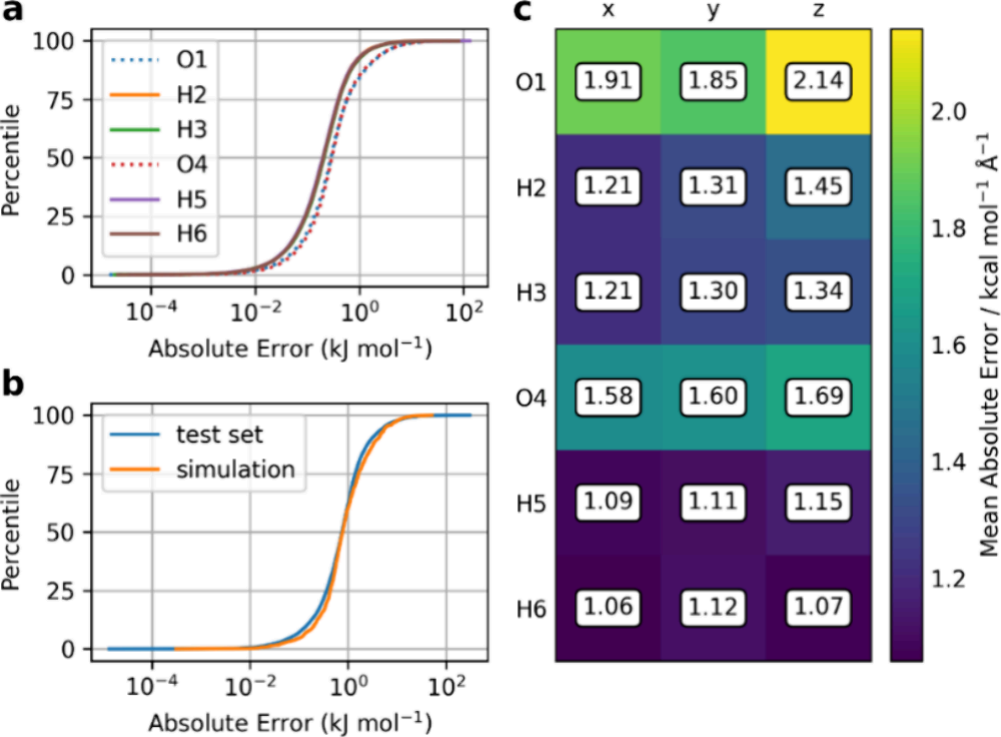
(a) Atomic S-curves of IQA energy for the 15,000-point test set. (b) Total system energy S-curves for 15,000 test sets and 1000 random geometries from a 298 K FFLUX simulation. (c) Atomic forces MAE plot for each Cartesian coordinate, averaged over 1000 geometries from a 298 K FFLUX simulation.

The prediction accuracy of the GPR models is benchmarked by both the independent test set as well as by FFLUX molecular dynamics simulations, the results of which are shown in Figure 4. The atomic energy prediction accuracy for the test set is monitored with S-curves, which plot the absolute prediction error of a property against the (cumulative) percentile of geometries displaying this error or less. A very similar performance can be seen across the set of four hydrogen atoms and the set of two oxygen atoms. For example, the mean absolute error (MAE) of the hydrogen atoms is 0.41 (H2), 0.47 (H3), 0.43 (H5), and 0.42 (H6) kJ mol^–1^. The hydrogen atoms do have better performance than the heavier oxygen atoms because the S-curves are shifted to the left, indicating a lower prediction error. This result is most likely due to a combination of factors such as higher coordination numbers and larger atomic volumes of heavier atoms, which increase numerical noise in calculations. The similar prediction performance for each type of atom was expected due to the symmetry of the water dimer that is captured by the WD24 data set. The two water molecules are virtually indistinguishable from one another in the data set, meaning that model performance should also be very similar. Since models are also atom based, there is a separate set of hyperparameters associated with each atom. Therefore, predictions will differ slightly for chemically identical atoms.

Next, the accuracy of the total system energy predictions is checked for both the test set and in simulation. Figure 4b shows the S-curves for the total system energy prediction errors of both the test set, as well as 1000 geometries taken from a 298 K FFLUX simulation with the 65,000-point models. The MAE of the total energy for the test set is 1.69 kJ mol^–1^, while the MAE for the simulation is 1.75 kJ mol^–1^. Roughly 60% of water dimer geometries, in both the test set and in simulation, have the total energy predicted within 1 kJ mol^–1^ and around 90% predicted within 1 kcal mol^–1^, which is often considered within chemical accuracy. The similar model performance on the test set and in the simulation suggests that the test set performance is a reliable indicator for simulation performance. It is also interesting to note that the simulation geometries have a lower maximum prediction error compared to the test set. This indicates that the test set contains more distorted geometries than what may be seen in simulation.

The atomic force prediction accuracy is also assessed by calculating the MAE of each force component as the average over the extracted 1000 simulation geometries, the results of which are shown in Figure 4c. Since GPR models are trained for each atom, the optimized hyperparameters will be different, and therefore the model predictions will be different. Similarly to the case of the IQA energy S-curves, chemically identical atoms will have different force predictions. The hyperparameters do play an important role in the forces because different sets of hyperparameters for each atomic model will change the gradient of the PES that the GPR model fits, and consequently the force predictions. The variability of accuracies observed between the *x*, *y*, and *z* components is also due to the hyperparameters, as each input dimension of the GPR models has its own hyperparameters. The hydrogen atoms have a lower MAE for all force components when compared to the oxygen atoms for the same reasons as in the S-curves discussed previously.

**Figure 5:**
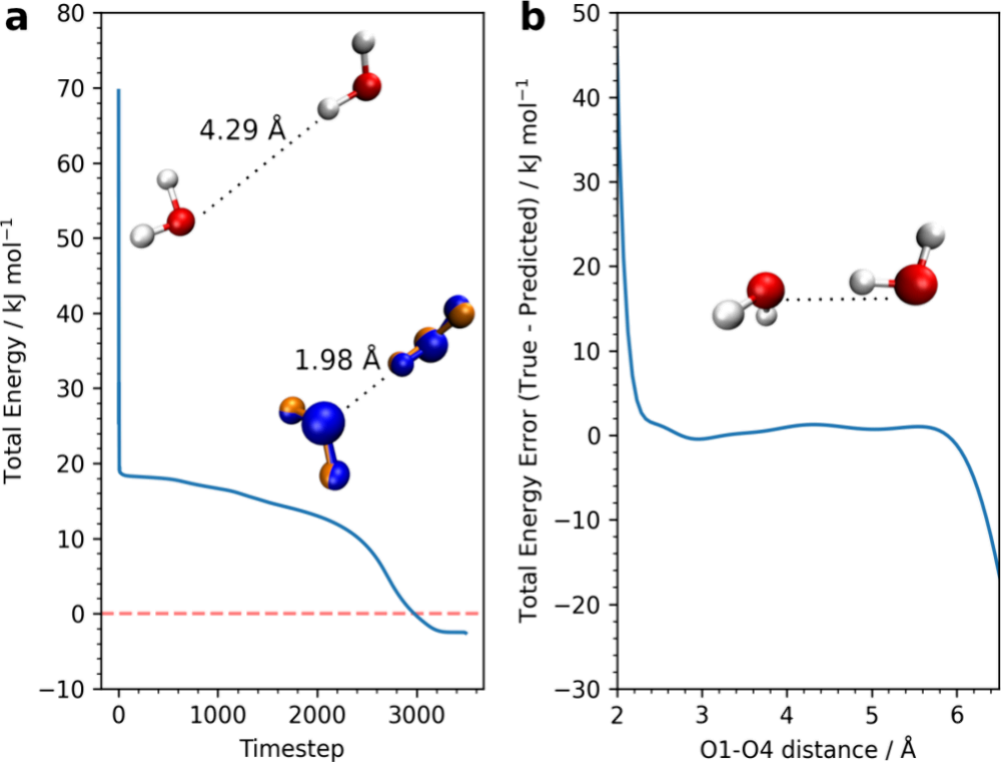
(a) Water dimer optimization from a starting geometry (top-left) using 65,000 training point GPR models. The overlapped FFLUX optimized geometry (orange) and the true global minimum geometry (blue) are shown in the bottom right. The shown distance for the optimized structure is obtained by the FFLUX simulation. The true total energy of the global minimum is subtracted such that the dashed red line at 0 kJ mol^–1^ indicates the true total energy minimum. (b) Total energy prediction error for scan along the O1–O4 distance vector. The starting geometry used is the global minimum geometry. The dotted line indicates the scan coordinate.

For the purpose of additional model testing, a geometry optimization and a coordinate scan were performed, the results of which are shown in Figure 5. The starting dimer geometry used for the optimization has a O···H distance of nearly 4.3 Å between the two water molecules, which is very far from the known global minimum geometry, which has a distance of 1.95 Å. The FFLUX optimization, guided only by the GPR-predicted energies and forces, converges in 3496 timesteps to a minimum which is 2.6 kJ mol^–1^ below the true global minimum total energy, and is within the threshold of chemical accuracy. Note that it is possible for the model to predict an energy that is lower than the true minimum energy because the GPR model has no knowledge of the global minimum geometry because it is not part of the training data set. In fact, the lowest energy geometry found in the training set is around 9.5 kJ mol^–1^ above the global minimum energy (−152.9396793290 Ha). Distributions of the total system energies for the GPR training set can be found in Figures S4–S6 of the SI. The final FFLUX optimized geometry closely resembles the true global minimum geometry with a root-mean-square deviation (RMSD) of 0.09 Å between the two.

The scan along the O1–O4 coordinate was performed to check how well the shape of the PES is captured by the GPR models. The model accurately fits the PES of the water dimer in the range between 2.2 and 6.2 Å, with a total energy maximum absolute error of 4.1 kJ mol^–1^ in this distance range. The main reason why the model significantly deviates from the true total energy below 2.2 Å is the very high repulsion at very short distances. This repulsion leads to steep changes in the energy, which are difficult to capture by the machine learning models. It is interesting to note that the model does predict the total energy within chemical accuracy up to an O···O distance of around 6.0 Å, even though the training set contains O1–O4 distances of up to 5.5 Å. This finding indicates a degree of extrapolation outside of the training set. After 6.0 Å, predictions do start to deviate from the true values because the predictions return to the prior means of the GPR models when geometries very far away from training data are observed.

## Conclusions

To conclude, we presented a dimeric water dimer potential that will be used as a stepping stone for intrinsically capturing many-body interactions within the GPR models. The modeling procedure used in this work allows for training of much larger models that can be interfaced with FFLUX. The modeling procedure is general and can be applied to any chemical system. The ML potential was trained on the artificially generated WD24 data set, which uniformly samples the configuration space of the water dimer. The resulting GPR models trained on the WD24 data set can predict 90% of water dimer geometries seen in the test set and in 298 K FFLUX water dimer simulations within chemical accuracy. Additionally, the models are able to obtain an accurate optimized geometry with a total energy error of just 2.6 kJ mol^–1^, with a starting water dimer structure containing nearly 4.3 Å separation between the two water molecules. Since each atom has its own GPR model, the predictions between chemically identical atoms will be slightly different with the current modeling procedure. Additionally, the ALF features treat each atom uniquely, which does increase modeling complexity for a very flexible system such as the water dimer. The modeling complexity will grow as larger *N*-meric modeling is introduced within FFLUX, therefore the modeling architecture would need to be further enhanced to exploit the inherent symmetry that is present in water systems where possible. The addition of derivative information to the GPR models is already being explored to further improve performance.

### Computational Details

The WD24 data set consists of 100,000 geometries in total, with calculations done at the B3LYP/aug-cc-pVTZ level of theory. The data set is filtered and split into a 65,000-point training set and a 15,000-point test set per atom. The atomic IQA energy GPR models are trained on a single A100 NVIDIA GPU and model hyperparameters are optimized with the LBFGS optimizer. The FFLUX dimer molecular dynamics simulations were performed for 50 ps with 1 fs time step at 298 K using the *NVT* ensemble and Nosé–Hoover thermostat. Additional details of the data set generation and filtering process, modeling, and simulations can be found in Section 4 of the SI.

## Data Availability

The WD24 data set is available at https://github.com/popelier-group/WD24_dataset.

## Supporting Information Available

Additional details of computational chemistry programs and computational details; additional details on the GPR modeling procedure; details on the FFLUX definitions of the short-range and long-range interactions; WD24 data set generation process; plots of angular distributions of O4 atom; plots of WD24 total energy distributions; visualizations of MB-pol and q-AQUA data sets; and true and predicted PES plot along the O···O scan coordinate (PDF)
