# Supplementary material for: Modeling Many-Body Interactions in Water with Gaussian Process Regression
Source: J Phys Chem A. 2024 Oct 11;128(42):9345–51. doi: 10.1021/acs.jpca.4c05873 (PMC11514001; doi:10.1021/acs.jpca.4c05873)
Supplement: Supplementary file 1 — jp4c05873_si_001.pdf [file jp4c05873_si_001.pdf]

Supporting Information

Modelling Many-Body Interactions in Water with Gaussian Process Regression

Yulian T. Manchev and Paul L. A. Popelier*

Department of Chemistry, The University of Manchester, Manchester, M13 9PL, Great Britain

E-mail: paul.popelier@manchester.ac.uk

Contents

1. Additional Quantum Chemistry Program Details 2
2. Additional GPR Modelling Details 2
3. Additional Details on FFLUX Interactions 3
4. WD24 Dataset Generation Process 5
5. Dataset Angle Distributions 7
6. WD24 Dataset Energy Distributions 9
7. Water Dimer Dataset Visualisations 10
8. O1-O4 Coordinate Scan 13
9. FFLUX Simulation Settings 14
10. References 14

1. Additional Quantum Chemistry Program Details

The total system energy and wavefunctions for all geometries were calculated using GAUSSIAN16[1] at the B3LYP/aug-cc-pVTZ level of theory. All additional reference calculations by GAUSSIAN16, such as geometry optimisations and scans, were also performed at this level of theory. The resulting wavefunctions were fed into the AIMAll[2] program calculates the atomic energies and multipole moments.

2. Additional GPR Modelling Details

The sum of the atomic IQA energies, denoted E_{IQA}^{Ω}, over all atoms equals the total system energy E_{total}, as shown in Equation 1,

$$E_{total} = \sum_{\Omega}^{N_{atoms}} E_{IQA}^{\Omega} \tag{1}$$

Note that the program AIMAll performs numerical integration over the topological atom's volume, which means that there is a degree of numerical noise present in each E_{IQA}^{Ω}. This error is typically below 0.1 kJ mol^{-1} for most geometries. However, some geometries may contain very difficult atomic volumes to integrate over. Therefore, filtering based on integration error associated with AIMAll calculations is needed, as a high integration error leads to errors in values of both E_{IQA} and atomic multipole moments. The filtering applied to the WD24 dataset (discussed below in Section 4) ensures that only clean data is used for model training.

The predictions on the forces on atom Ω can be obtained analytically according to Equation 2,

$$F_c^{\Omega} = -\frac{\partial \hat{E}_{total}}{\partial c^{\Omega}} = -\sum_{A=1}^{N_{atoms}} \frac{\partial \hat{E}_{IQA}^{A}}{\partial c^{\Omega}} = -\sum_{A=1}^{N_{atoms}} \sum_{k=1}^{N_{features}} \frac{\partial \hat{E}_{IQA}^{A}}{\partial f_k^A} \frac{\partial f_k^A}{\partial c^{\Omega}} \tag{2}$$

where c indicates a global Cartesian coordinate (x, y or z direction). The partial derivatives $\frac{\partial f_k^A}{\partial c^{\Omega}}$ related the ALF descriptors to the global Cartesian coordinates and are purely based on the molecular geometry. Analytical expressions for the ALF descriptor derivatives have already been determined in a previous publication from our group.[3] The ALF descriptors are calculated by the ichor[4, 5] library. Note that the ALF features treat each atom uniquely, so permutations of the same types of atoms are not captured within the current GPR models.

All models used in the current work used the conjugate gradients (CG) approach, as implemented by GPyTorch to obtain the GPR weights, instead of Cholesky decomposition. A preconditioner size of 100 was used for the models, and the CG convergence tolerance was set to 0.01 for training the models. The LBFGS optimiser was used to optimise the model hyperparameters through the "marginal log likelihood" function. The GPR models are trained in a sequence of steps, which ensures that optimal hyperparameters are reached in a timely manner, as increasing the training set size does increase computational cost. First, a subset of the full training set is created by random sampling, and GPR models are only trained with 5,000 training points. The optimal hyperparameters found for the 5,000-point models are used as the starting hyperparameters of GPR models with 20,000-training-set points. Finally, the optimal hyperparameters of the 20,000-point models are fine-tuned for the 65,000-training-point models. The maximum possible optimiser iterations for the 5,000- and 20,000-point models was set to 1,000, and 20 for the 65,000-point model. Note that the LBFGS optimiser can converge before the maximum number of iterations is reached because of line-search convergence criteria. The higher number of iterations for the 5,000 and 20,000-point models was set because of their lower computational cost per optimiser iteration. As the number of training points increases, the number of optimiser iterations becomes the most costly factor in model training. Therefore, for larger datasets a much smaller number of optimiser iterations is set. Training sets of 65,000 points are used for the IQA energy GPR models of each atom. The size of the training set presents an astronomical jump in the number of training points of GPR models used within FFLUX, which have so far used around 8,000 training points at most. The test sets used as one the benchmarks for the models' predictive accuracy is also significantly higher at 15,000 points.

3. Additional Details on FFLUX Interactions

Interactions are split into short-range and long-range, and the total system energy is defined as the sum of the two, as shown in Equation 1. Short-range interactions are directly defined as the sum of predicted IQA energies, $E_{\mathrm{IQA}}^{\mathrm{A}}$, over all atoms A as shown in Equation 2. On the other hand, long-range interactions are a combination of electrostatic and van der Waals interactions, namely $E_{\mathrm{electrostatic}}$ and E_{vdw}, as shown in Equation 3. GPR models are also involved in long-range interactions because they can be trained to predict polarisable atomic multipole moments, which are subsequently used to calculate $E_{\mathrm{electrostatic}}$.

$$E_{\text{total}} = E_{\text{sr}} + E_{\text{lr}} \tag{3}$$

$$E_{\text{sr}} = \sum_{\text{A}} E_{\text{IQA}}^{\text{A}} \tag{4}$$

$$E_{\text{lr}} = E_{\text{electrostatic}} + E_{\text{vdw}} \tag{5}$$

In monomeric modeling, the GPR models are trained on properties of a single molecule, without any knowledge of the environment outside of that molecule. With monomeric modelling, short-range interactions are defined to be all interactions within a single molecule. Consequently, interactions between the two water molecules in the water dimer are always considered long-range and are included through high-order multipolar electrostatics and van der Waals interactions. Monomeric modelling has already been used for condensed phase water simulations, where FFLUX has shown to perform on a par with other state-of-the-art force fields. The modelling procedure is transferable to larger water systems where each monomer utilises the exact same set of GPR models for atomic IQA energies and multipole moments. However, the model predictions will be different for each monomer as they depend on the molecular configuration.

In dimeric modelling, the GPR models inherently capture all interactions between the two molecules, and a more realistic representation of an atomic environment is achieved, which transfers to more accurate simulations. When the water dimer system is treated with dimeric modelling, all interactions are considered purely short-range. Long-range interactions only come into play when modelling a system larger than the water dimer. For example, if a water tetramer is being modelled, it can be split into two sets of water dimers. The atomic IQA energies and multipole moments associated with individual dimers can be predicted separately with dimeric GPR models. All interactions in each water dimer will be considered short-range but interactions between the two water dimers will be considered long-range. Similar to the monomeric modelling case, each water dimer system can utilise the same set of dimeric GPR models and dimeric modelling can also be used for treatment of larger water clusters or box simulations. The dimeric models serve as a stepping stone to *N*-meric modelling and provide a more accurate representation of local environments of atoms than monomeric modelling.

4. WD24 Dataset Generation Process

To generate each geometry in the dataset, two water molecules (containing atoms O1, H2, H3 and O4, H5, H6, respectively) are generated with random O-H bond lengths and H-O-H angles, and placed in the *xy*-plane. The oxygen atom of both molecules is placed at the origin (0,0,0) such that the two water molecules are sitting on top of each other in the xy plane. The molecule containing atoms O4, H5, H6 is then rotated using a random rotation matrix, and then translated in a random direction within a certain distance threshold. After this this process the O4 atom is no longer at (0,0,0) but the O1 atom is. The translation happens by adding the coordinates of vectors that have been sampled from a uniform unit sphere and scaled to have a magnitude between 2.0 Å and 5.5 Å. The O-O distance is in the range of 2.0 Å and 5.5 Å and was specifically chosen to cover the range of O-O distances found in the first two solvation shells of a water molecule in liquid water.[6] The O-H bond length in each water molecule is in the range of 0.85 Å and 1.1 Å, and the H-O-H angle between 90° and 115°. A total of 100,000 geometries was generated, and more distortions and larger distances between molecules can be readily generated.

Multiple filtering steps are applied to the WD24 dataset to ensure the quality of the resulting atomic training sets. Geometries containing an absolute integration error higher than a rather generous 0.001 a.u. for any of the atoms are removed. Geometries for which the total sum of the atomic IQA energies exceeds 1 kJ mol^{-1} when compared to the total system energy are also filtered out. These two filtering steps remove roughly 100 geometries for each atom. This loss represents a very small portion of the total dataset size but it is critical that any outliers are removed before model training. In theory, there should not be any outliers in *ab initio* data but, in practice, outliers are present due to numerical integration behind obtaining the IQA data. An additional filtering step ensures that the molecular geometries used to train each atom are the same because each atom has its own dataset. Note that, although the geometries used in the training sets are the same across all atoms, the actual ALF descriptors used to represent each atom will be different. This final filtering step leaves 87,711 geometries, which are split into training and test sets by random sampling without replacement to ensure that each point is only added once to the training set. The test set does not contain any geometries included in the training set.

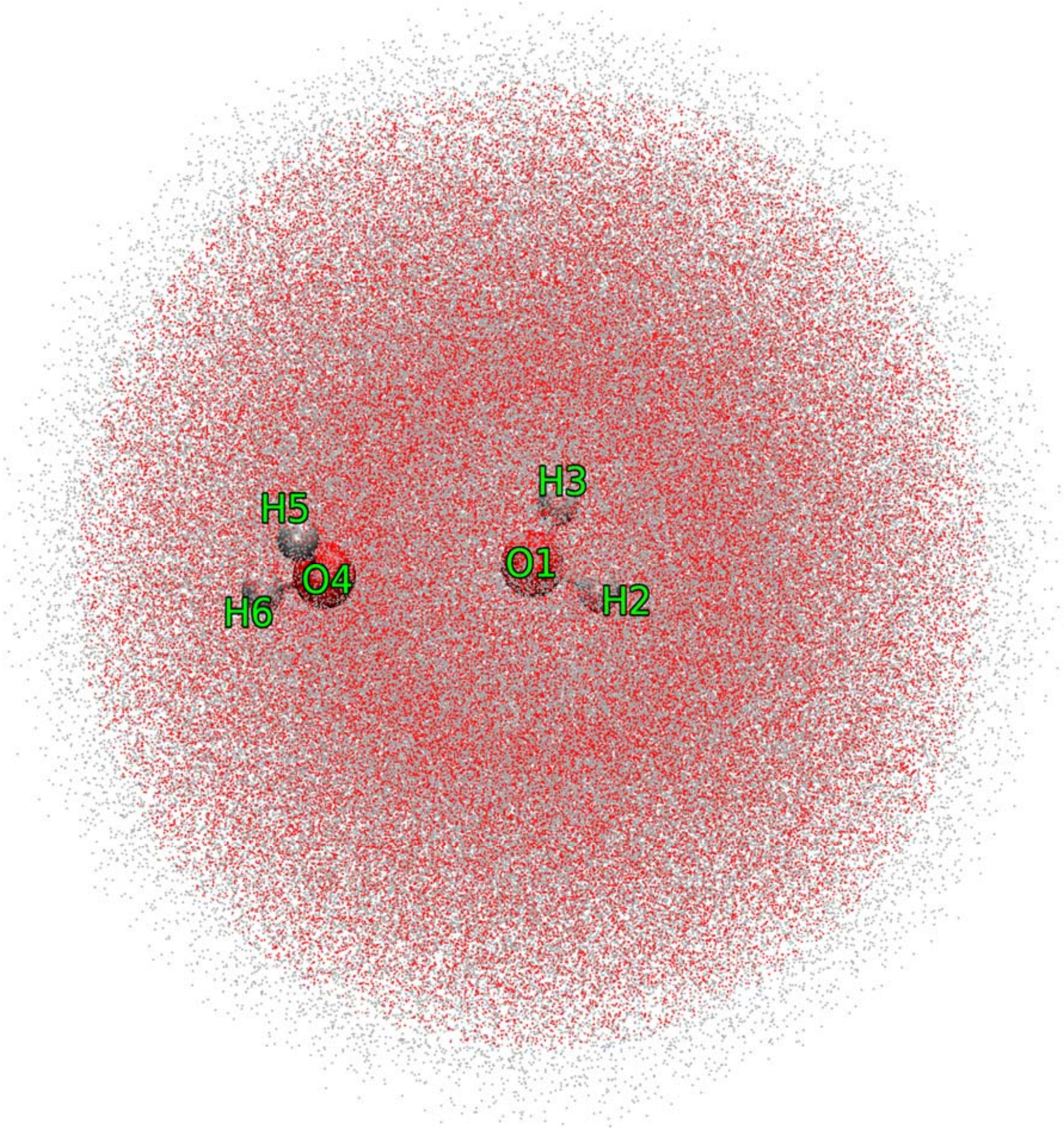

Figure S1. Visual representation of all water dimer geometries in the WD24 dataset, created using the VMD[7] program. One example water dimer geometry is shown by means of the "CPK" representation, while all other geometries are shown via the "points" representation. Grey points indicate hydrogens and red points indicate oxygens.

5. Dataset Angle Distributions

As discussed in the main text, the MB-pol so-called “short-range" dataset consists of 42,508 geometries gathered from four separate datasets: CC-pol, pimd-run105, pimd-run107, and stationary datasets. The q-AQUA dataset is a combination of the MB-pol dataset as well as the HBB2 dataset, and consists of 71,892 geometries. Figure S1 shows the distribution of the O4 θ ALF feature (the polar angle) calculated with the ALF centered on O1, with x-axis atom H2 and xy-plane atom H3. Figure S2 shows the distribution of the O4 ϕ ALF feature (the azimuthal angle) calculated with the ALF centered on O1, with x-axis atom H2 and xy-plane atom H3.

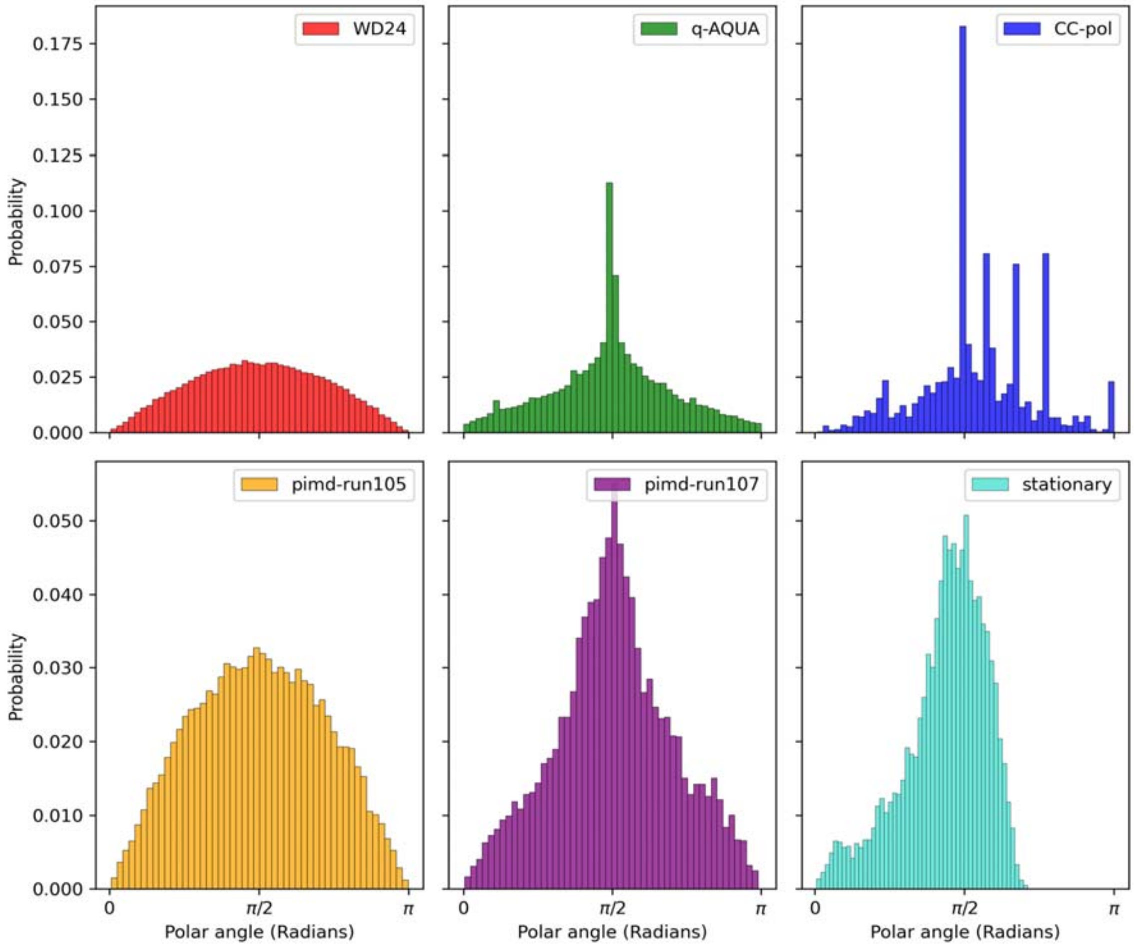

Figure S2. Distribution of O4 θ ALF feature calculated with ALF centered on O1, with x-axis atom H2, and xy-plane atom H3. The z-axis is calculated to be perpendicular to the xy-plane. This is the polar angle that the O4 atom creates with the local axis system.

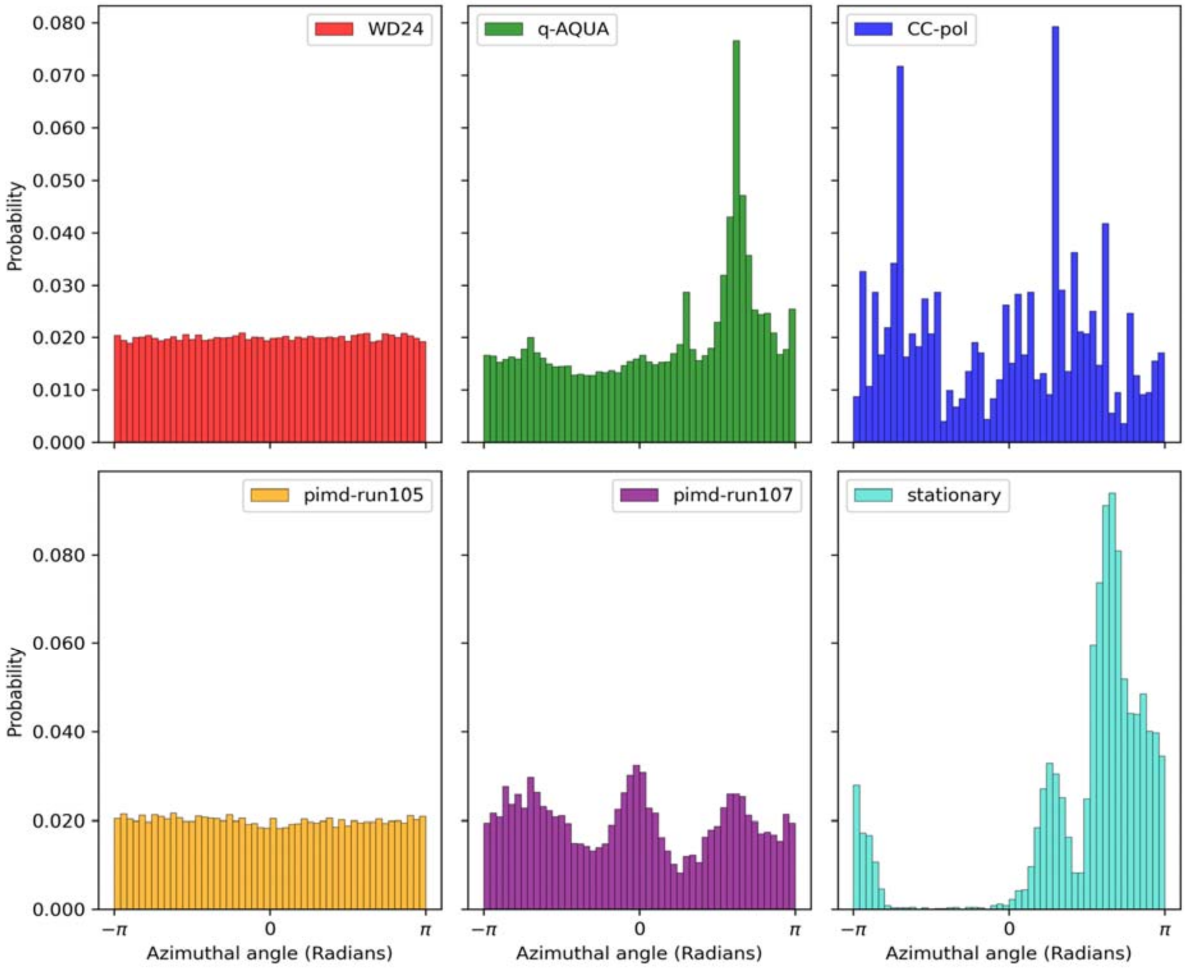

Figure S3. Distribution of the O4 ϕ ALF feature calculated with the ALF centered on O1, with x-axis atom H2 and xy-plane atom H3. The z-axis is calculated to be perpendicular to the xy-plane. This is the azimuthal angle ϕ that the O4 atom creates with the local axis system.

6. WD24 Dataset Energy Distributions

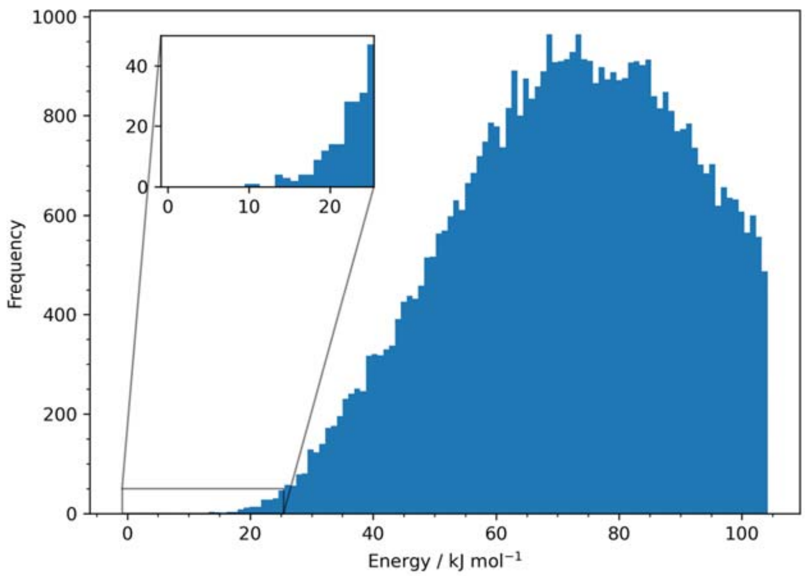

Figure S4. Energy distribution of the 65,000-point training set, shown with an energy cutoff of roughly 100 kJ mol^{-1} above the known global minimum energy (at B3LYP/aug-cc-pVTZ level of theory). The energy of the known global minimum geometry (-152.9396793290 Ha) (geometry shown in Figure 5 of the main text) is subtracted and units are converted to kJ mol^{-1}. The zoomed-in region (see inset) shows the number of geometries that have energies closest to the global minimum energy.

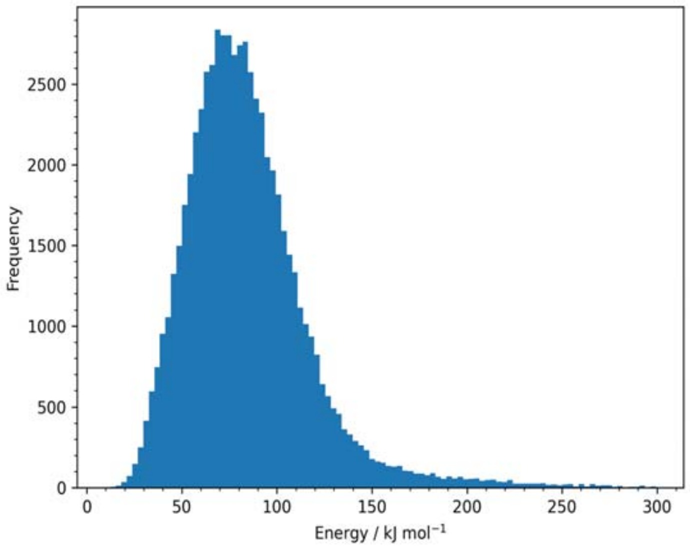

Figure S5. Energy distribution of the 65,000-point training set but truncated because of an energy cutoff set at 300 kJ mol^{-1} above the known global minimum energy (at B3LYP/aug-cc-pVTZ level of theory). The energy of the known global minimum geometry (-152.9396793290 Ha) is subtracted and units are converted to kJ mol^{-1}.

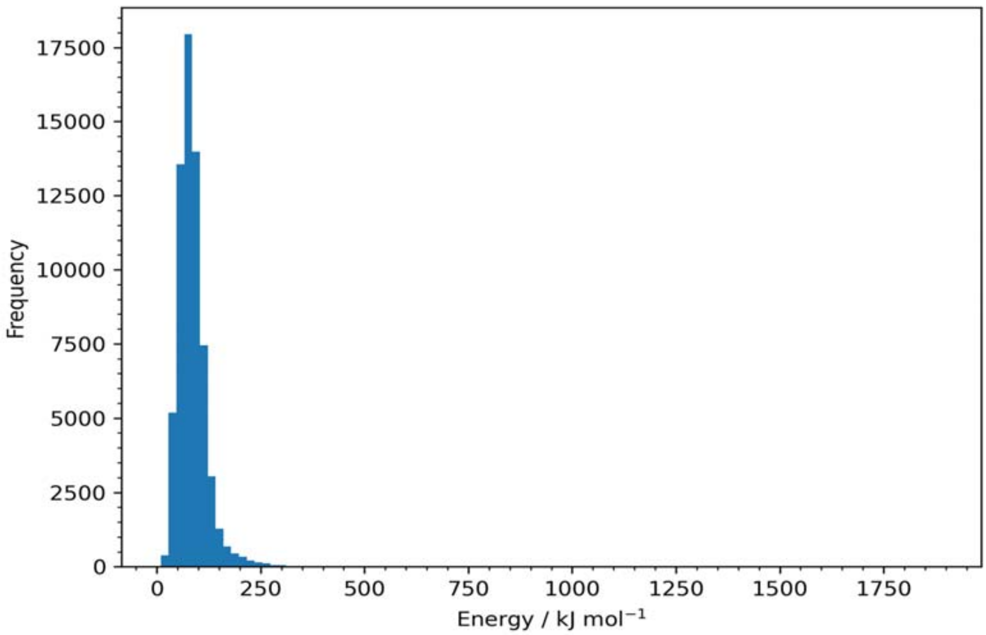

Figure S6. Energy distribution of the **full** 65,000-point training set without energy cutoff. The energy of the known global minimum geometry (-152.9396793290 Ha) is subtracted and units are converted to kJ mol^{-1}. There are a few geometries with very high energies but they cannot be easily spotted.

7. Water Dimer Dataset Visualisations

All figures below were generated by calculating the ALF features of the O1 atom (with H2 and H3 atoms as the x-axis and xy-plane atoms) for all geometries in the given dataset, and then converting the features back into Cartesian coordinates. This effectively centers all geometries with the origin being the O1 atom (and H2 atom moving in x-axis and H3 atom moving in xy-plane). The position of all other atoms is mapped with respect to this local coordinate system. These geometries can then all be overlapped to obtain a visual representation of the dataset and check which configurations are present in the dataset. A region with higher point density indicates that more geometries are present there.

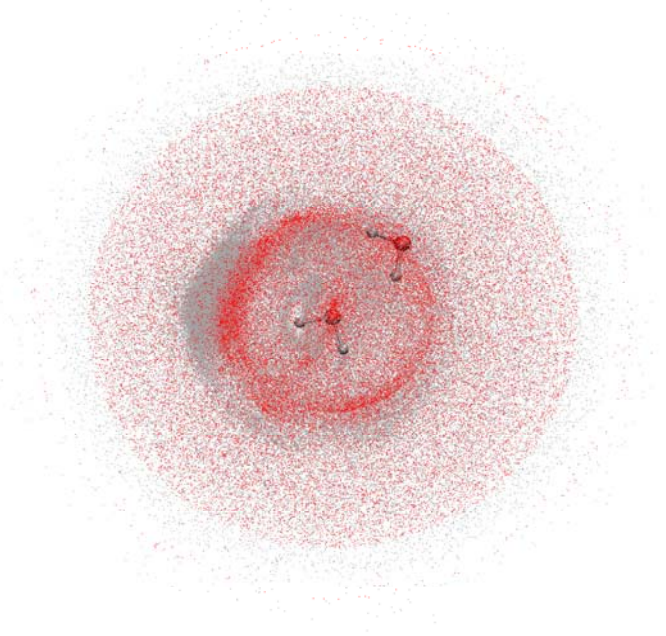

Figure S7. Visualisation of the q-AQUA water dimer dataset.

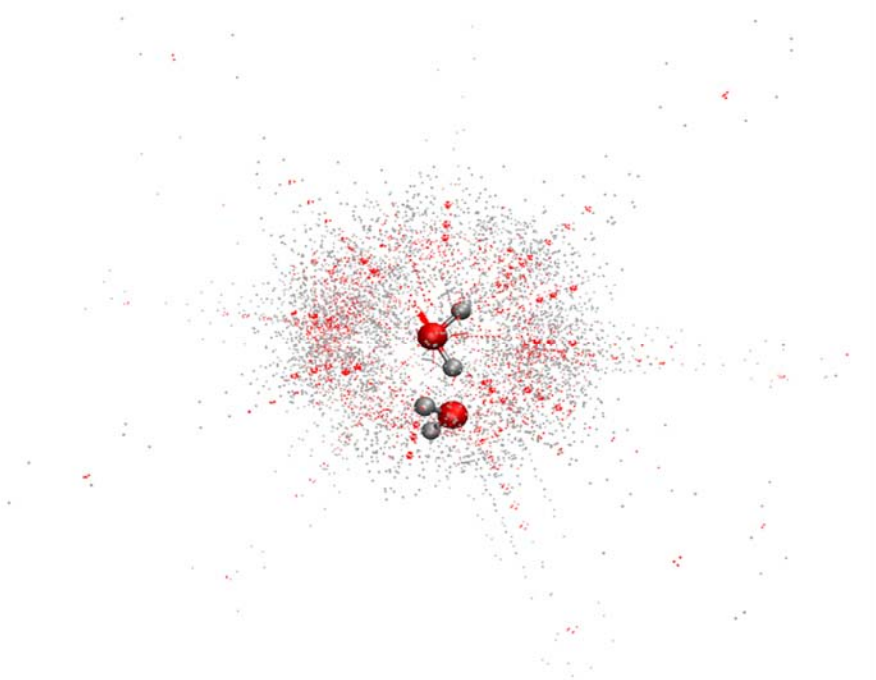

Figure S8. Visualisation of the CC-pol water dimer dataset, which is part of the MB-pol training dataset.

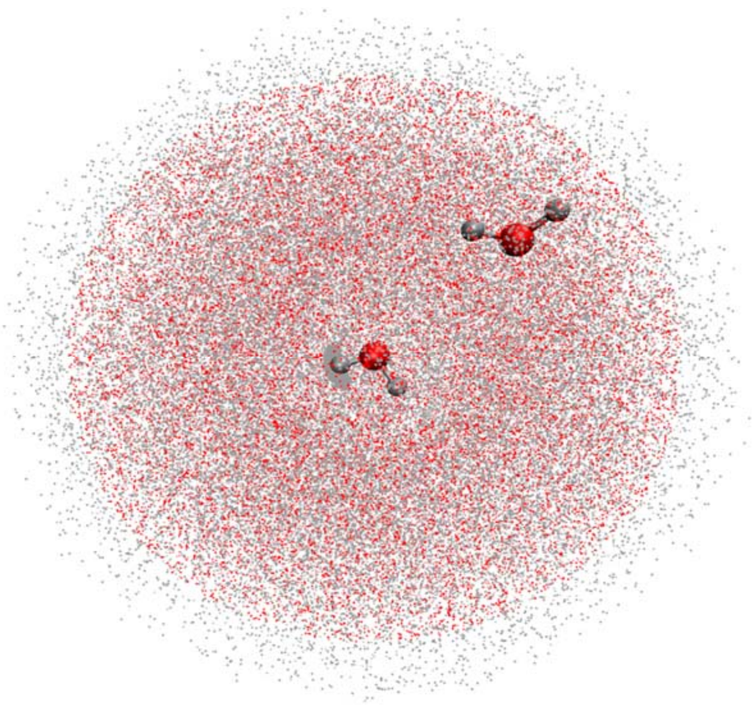

Figure S9. Visualisation of the HBB2 pimd-run105 water dimer dataset, which is part of the MB-pol training dataset.

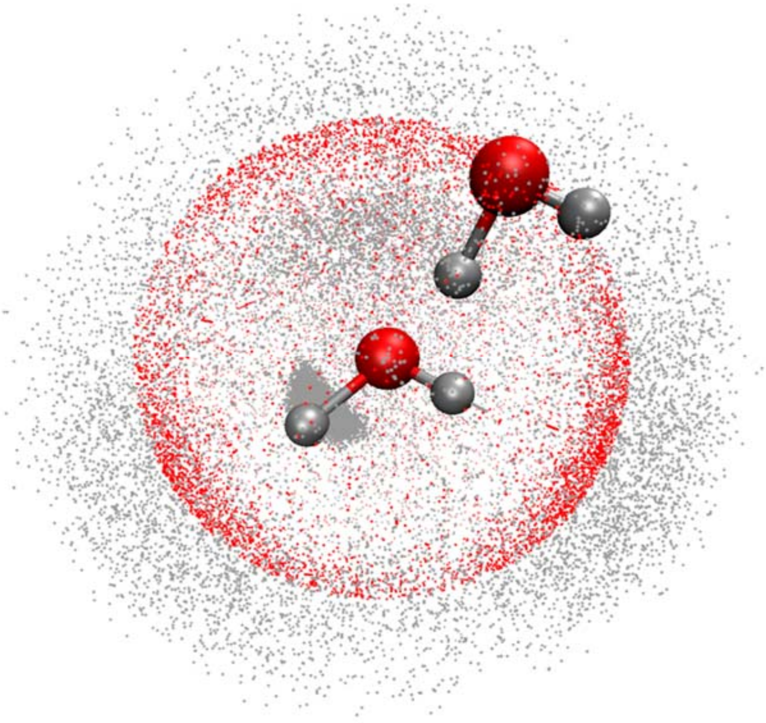

Figure S10. Visualisation of the HBB2 pimd-run107 water dimer dataset, which is part of the MB-pol training dataset.

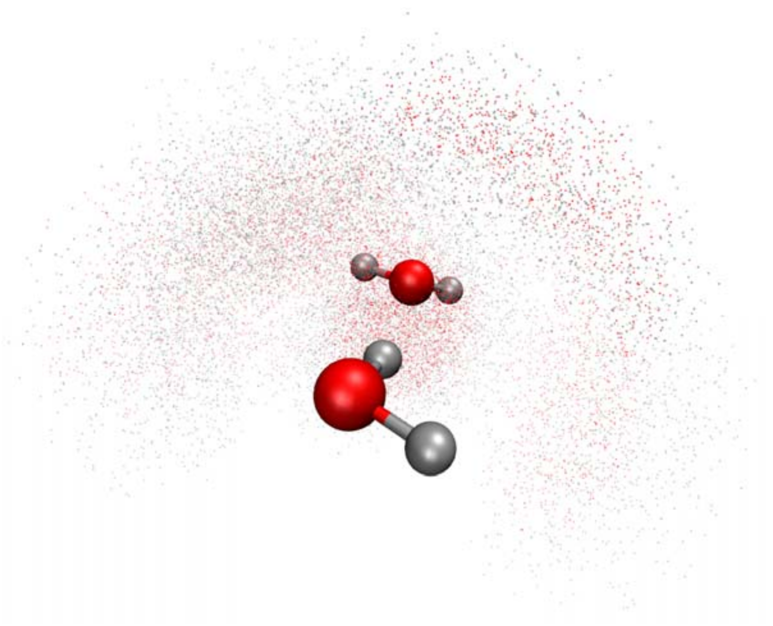

Figure S11. Visualisation of the stationary water dimer dataset, which is part of the MB-pol training dataset.

8. O1-O4 Coordinate Scan

The scan was performed along the O1-O4 coordinate, starting from the true global minimum geometry. The distances were scanned from 2 Å to 6.5 Å.

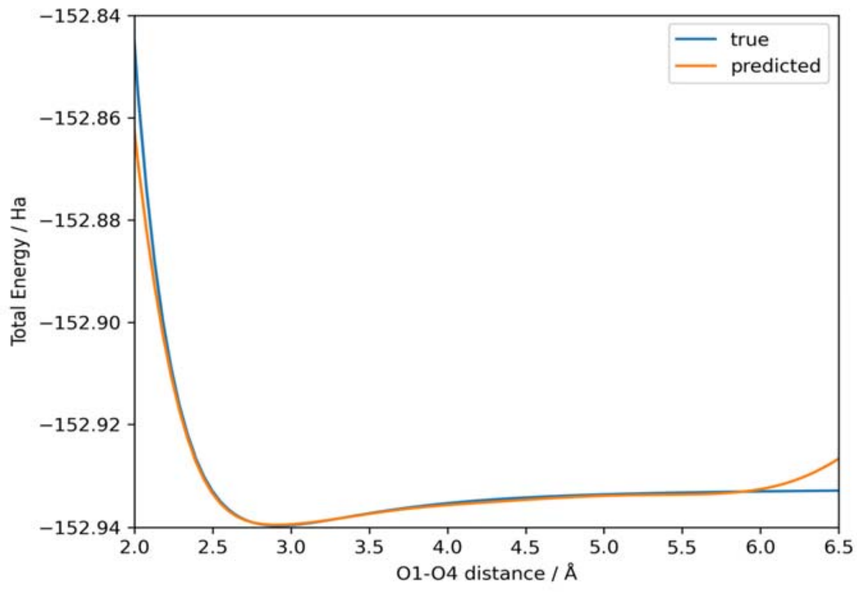

Figure S12. True and predicted total system energies along O1-O4 scan.

9. FFLUX Simulation Settings

The 1000 geometries used to benchmark model predictions were picked randomly from a 50 ps simulation FFLUX simulation performed at 298 K, with a timestep of 1 fs using NVT ensemble and the Nosé-Hoover thermostat. The true total energies (and forces) were calculated at the B3LYP/aug-cc-pVTZ level with GAUSSIAN16, for comparison against the model predictions. The geometry optimisation was performed at 1 K temperature with the "distance" criterion set to 0.000001. The final FFLUX geometry was taken and optimised with GAUSSIAN16 (at the B3LYP/aug-cc-pVTZ level of theory) for comparison.

10. References

(1) *Gaussian16 Rev. C.01*; Gaussian, Inc.: Wallingford, CT, 2016.

(2) *AIMAll*; TK Gristmill Software: Overland Park, KS, USA, 2019.

(3) Mills, M. J. L.; Popelier, P. L. A. Electrostatic Forces: Formulas for the First Derivatives of a Polarizable, Anisotropic Electrostatic Potential Energy Function Based on Machine Learning. *Journal of Chemical Theory and Computation* **2014**, *10* (9), 3840-3856.

(4) Burn, M. J.; Popelier, P. L. A. ICHOR: a modern pipeline for producing Gaussian process regression models for atomistic simulations. *Materials Advances* **2022**, *3* (23), 8729-8739.

(5) *ichor: Computational Chemistry Data Management Library for Machine Learning Force Field Development*; GitHub: Manchester, UK, 2024. https://github.com/popelier-group/ichor.

(6) Soper, A. K. The radial distribution functions of water and ice from 220 to 673 K and at pressures up to 400 MPa. *Chemical Physics* **2000**, *258* (2), 121-137.

(7) Humphrey, W.; Dalke, A.; Schulten, K. VMD: Visual molecular dynamics. *Journal of Molecular Graphics* **1996**, *14* (1), 33-38.
